# *CASC15* gene polymorphisms reduce neuroblastoma risk in Chinese children

**DOI:** 10.18632/oncotarget.20514

**Published:** 2017-08-24

**Authors:** Jiao Zhang, Zhen-Jian Zhuo, Jiaxiang Wang, Jing He, Lin Yang, Da Zhang, Pan Qin, Lizhao Yan

**Affiliations:** ^1^ Department of Pediatric Surgery, The First Affiliated Hospital of Zhengzhou University, Zhengzhou 450052, Henan, China; ^2^ Department of Pediatric Surgery, Guangzhou Institute of Pediatrics, Guangzhou Women and Children’s Medical Center, Guangzhou Medical University, Guangzhou 510623, Guangdong, China; ^3^ School of Chinese Medicine, Faculty of Medicine, The Chinese University of Hong Kong, Hong Kong 999077, China

**Keywords:** neuroblastoma, CASC15, 6p22, polymorphism, susceptibility

## Abstract

In this case-control study, we analyzed the association between three single nucleotide polymorphisms (SNPs) in the *CASC15* gene (rs6939340 A>G, rs4712653 T>C, and rs9295536 C>A) and neuroblastoma susceptibility in the Guangdong and Henan populations of China. We genotyped and analyzed 118 cases and 281 control subjects from Henan province and combined them with previously published data from the Guangdong population. In the Henan population, only the rs6939340 G>A variant homozygote AA was associated with decreased neuroblastoma risk [AA vs. GG: adjusted odds ratio (OR) = 0.47, 95% confidence interval (CI) = 0.23-0.98; *P*=0.045]. All three polymorphisms, individually and in combination, were associated with decreased neuroblastoma susceptibility in the Guangdong population. Moreover, subjects carrying 1-3 of these protective genotypes had lower neuroblastoma susceptibility than non-carriers (adjusted OR=0.65, 95% CI=0.51-0.84, *P*=0.0007). These results show that all three genetic variants of *CASC15* identified in a genome-wide association study (GWAS) decrease neuroblastoma risk in two distinct Chinese populations.

## INTRODUCTION

Neuroblastoma is an embryonal cancer that arises from primordial sympathetic neural precursors in infants [1]. The incidence rate of neuroblastoma is about 1 in 7000 in the USA [2] and 7.7 per million in China [3]. Neuroblastoma is the most common solid tumor in childhood and despite marked improvements in its treatment, it still accounts for 15% of the cancer-related mortality in children [4]. Neuroblastoma is characterized by diverse clinical behaviors based on which it is classified into low risk, intermediate risk and high risk groups [5]. Typically, high-risk patients have widely disseminated disease at diagnosis and poor survival rates, whereas most of the low-risk patients spontaneously regress without chemotherapy [6, 7]. Nearly 50% of the neuroblastoma patients are diagnosed as high-risk and less than 40% of them survive despite intensive therapies [4, 8]. Moreover, survivors face lifelong serious co-existing health issues that affect their social life [9].

Only 1% neuroblastoma cases are familial, and in most cases are associated with *ALK* gene mutations [10, 11]. However, the exact etiology of sporadic neuroblastoma is obscure [1]. Extrinsic risk factors that influence neuroblastoma like exposure to radiation and other environmental factors have not yet been identified. Few children develop neuroblastoma because their parents are exposed to radiation sources, wood dust and hydrocarbons [12, 13]. Therefore, genetic factors combined with environmental factors can influence neuroblastoma outcomes.

The availability of the human genome sequence and high density single nucleotide polymorphism (SNP) arrays have resulted in genome-wide association studies (GWASs), which shed more light into possible genetic mechanisms of human diseases including cancers [14]. GWAS on neuroblastoma identified several susceptibility loci. SNPs in *HSD17B12*, *DUSP12*, *IL31RA* and *DDX4* genes were enriched in the low-risk group of patients [15]. SNPs in *BARD1* [16], cancer susceptibility candidate 15 (*CASC15*) [17], and *LMO1* [18] genes were enriched in high-risk neuroblastoma patients. Also, some common variants were associated with neuroblastoma risk, but were not correlated with the disease phenotype [19]. Since its possible to miss relevant risk variants because of stringent threshold parameters in GWAS studies, alternative approaches were used to identify potential variants associated with neuroblastoma including *NEFL* [20] and *TP53* [21]. Most GWAS-identified SNPs in neuroblastoma have been verified in other population samples [22-24]. Yet, only four studies have assessed the association of polymorphisms in *CASC15* gene with neuroblastoma risk [22-25].

In our previous study, we reported that *CASC15* gene polymorphisms were associated with neuroblastoma risk in Southern Chinese population [25]. In this study, we evaluate the association between *CASC15* gene polymorphisms and neuroblastoma risk in two combined cohorts of neuroblastoma patients from Northern China.

## RESULTS

### Population characteristics

The detailed demographic characteristics of Guangdong population were reported previously [25-27]. For Henan population, a summary of demographic characteristics of the 118 cases and 281 controls are shown in Table 1. The mean age at diagnosis was 46.24 ± 29.98 months for the cases (range 0 to 131.1 months) and 44.97 ± 33.23 months for the controls (range 0.1 to 144.0 months). There were no differences in age and gender (*P*=0.189 and 0.196, respectively) between the case and control subjects. According to the INSS criteria [28], 15 (12.82%), 31 (26.50%), 19 (16.24%), 49 (41.88%), and 3 (2.56%) cases were classified into stages I∼V, and 4s, respectively. Out of the 118 cases, adrenal gland, mediastinum and other regions accounted for 89 (75.42%), 19 (16.10%), and 10 (8.47%) neuroblastomas, respectively.

**Table 1:** Frequency distribution of selected characteristics in neuroblastoma cases and cancer-free controls

Variables	Cases (n=118)		Controls (n=281)		*P* ^*a*^
	No.	%	No.	%	
Age range, month	0-131.1		0.1-144.0		0.189
Mean ± SD	46.24 ± 29.98		44.97 ± 33.23		
≤18	23	19.49	72	25.62	
>18	95	80.51	209	74.38	
Gender					0.196
Female	54	45.76	109	38.79	
Male	64	54.24	172	61.21	
Clinical stages					
I	15	12.82			
II	31	26.50			
III	19	16.24			
IV	49	41.88			
4s	3	2.56			
Sites of origin					
Adrenal gland	89	75.42			
Mediastinum	19	16.10			
Other regions	10	8.47			

^**a**^Two-sided *χ*^*2*^test for distributions between neuroblastoma cases and cancer-free controls.

### Association between *CASC15* gene polymorphisms and neuroblastoma susceptibility

Table 2 shows the genotype frequencies of the *CASC15* gene polymorphisms in the cases and controls from Henan and Guangdong provinces, both separately and combined, and their association with neuroblastoma risk. The three polymorphisms, rs6939340 G>A (*P*=0.970), rs4712653 C>T (*P*=0.290) and rs9295536 A>C (*P*=0.287) were in accordance with Hardy-Weinberg equilibrium (HWE) for combined subjects. In the case subjects from Henan province, we found decreased neuroblastoma risk with the rs6939340 AA genotype compared to the wild-type GG genotype (AA vs. GG: adjusted OR=0.47, 95% CI=0.23-0.98, *P*=0.045). However, the two other genotypes, rs4712653 C>T and rs9295536 A>C were not associated with neuroblastoma risk.

**Table 2:** Association between *CASC15* gene polymorphisms and neuroblastoma susceptibility

Genotype	Henan province				Guangdong province				Combined			
	Cases (N=118)	Controls (N=281)	Adjusted OR (95% CI) ^a^	*P*^a^	Cases (N=255)	Controls (N=531)	Adjusted OR (95% CI) ^a^	*P*^a^	Cases (N=373)	Controls (N=812)	Adjusted OR (95% CI) ^a^	*P*^a^
rs6939340 G>A (HWE=0.970) ^b^												
GG	58 (49.15)	115 (40.93)	1.00		155 (60.78)	232 (43.69)	1.00		213 (57.10)	347 (42.73)	1.00	
AG	49 (41.53)	121 (43.06)	0.80 (0.50-1.26)	0.336	81 (31.76)	247 (46.52)	**0.49 (0.36-0.68)**	<0.0001	130 (34.85)	368 (45.32)	**0.58 (0.44-0.75)**	<0.0001
AA	11 (9.32)	45 (16.01)	**0.47 (0.23-0.98)**	0.045	19 (7.45)	52 (9.79)	**0.55 (0.31-0.96)**	0.036	30 (8.04)	97 (11.95)	**0.50 (0.32-0.78)**	0.0024
Additive			**0.72 (0.53-0.99)**	0.046			**0.61 (0.48-0.79)**	<0.0001			**0.65 (0.54-0.79)**	<0.0001
Dominant	60 (50.85)	166 (59.07)	0.71 (0.46-1.10)	0.121	100 (39.22)	299 (56.31)	**0.50 (0.37-0.68)**	<0.0001	160 (42.90)	465 (57.27)	**0.56 (0.44-0.72)**	<0.0001
Recessive	107 (90.68)	236 (83.99)	0.53 (0.26-1.06)	0.073	236 (92.55)	479 (90.21)	0.74 (0.43-1.28)	0.286	343 (91.96)	715 (88.05)	**0.64 (0.42-0.99)**	0.044
rs4712653 C>T (HWE=0.290) ^b^												
CC	69 (58.47)	154 (54.80)	1.00		171 (67.06)	285 (53.67)	1.00		240 (64.34)	439 (54.06)	1.00	
CT	41 (34.75)	99 (35.23)	0.93 (0.58-1.48)	0.754	69 (27.06)	209 (39.36)	**0.55 (0.39-0.77)**	0.0004	110 (29.49)	308 (37.93)	**0.65 (0.50-0.86)**	0.0019
TT	8 (6.78)	28 (9.96)	0.64 (0.28-1.48)	0.298	15 (5.88)	37 (6.97)	0.68 (0.36-1.28)	0.230	23 (6.17)	65 (8.00)	0.65 (0.39-1.07)	0.088
Additive			0.85 (0.61-1.19)	0.352			**0.67 (0.52-0.87)**	0.0023			**0.73 (0.60-0.89)**	0.0023
Dominant	49 (41.53)	127 (45.20)	0.87 (0.56-1.34)	0.515	84 (32.94)	246 (46.33)	**0.57 (0.42-0.78)**	0.0004	133 (35.66)	373 (45.94)	**0.65 (0.51-0.84)**	0.0009
Recessive	110 (93.22)	253 (90.04)	0.66 (0.29-1.50)	0.318	240 (94.12)	494 (93.03)	0.84 (0.45-1.56)	0.581	350 (93.83)	747 (92.00)	0.75 (0.46-1.23)	0.260
rs9295536 A>C (HWE=0.287) ^b^												
AA	64 (54.24)	152 (54.09)	1.00		168 (65.88)	282 (53.11)	1.00		232 (62.20)	434 (53.45)	1.00	
AC	42 (35.59)	99 (35.23)	1.01 (0.63-1.61)	0.973	76 (29.80)	212 (39.92)	**0.60 (0.44-0.83)**	0.002	118 (31.64)	311 (38.30)	**0.71 (0.55-0.93)**	0.012
CC	12 (10.17)	30 (10.68)	0.96 (0.46-1.99)	0.903	11 (4.31)	37 (6.97)	0.50 (0.25-1.01)	0.054	23 (6.17)	67 (8.25)	0.64 (0.39-1.06)	0.081
Additive			0.99 (0.72-1.36)	0.940			**0.65 (0.50-0.84)**	0.001			**0.76 (0.62-0.93)**	0.0064
Dominant	54 (45.76)	129 (45.91)	1.00 (0.65-1.54)	0.985	87 (34.12)	249 (46.89)	**0.59 (0.43-0.80)**	0.0008	141 (37.80)	378 (46.55)	**0.70 (0.54-0.90)**	0.0050
Recessive	106 (89.83)	251 (89.32)	0.95 (0.47-1.94)	0.893	244 (95.69)	494 (93.03)	0.61 (0.30-1.21)	0.154	350 (93.83)	745 (91.75)	0.73 (0.45-1.19)	0.205
Combined effect of protective genotypes												
0	52 (44.07)	104 (37.01)	1.00		135 (52.94)	218 (41.05)	1.00		187 (50.13)	322 (39.66)	1.00	
1	15 (12.71)	49 (17.44)	0.60 (0.31-1.17)	0.133	35 (13.73)	67 (12.62)	0.84 (0.53-1.33)	0.454	50 (13.40)	116 (14.29)	0.74 (0.51-1.08)	0.118
2	5 (4.24)	11 (3.91)	0.92 (0.30-2.79)	0.879	19 (7.45)	11 (2.07)	**2.79 (1.29-6.06)**	0.009	24 (6.43)	22 (2.71)	**1.89 (1.03-3.46)**	0.040
3	46 (38.98)	117 (41.64)	0.78 (0.49-1.26)	0.315	66 (25.88)	235 (44.26)	**0.45 (0.32-0.64)**	<0.0001	112 (30.03)	352 (43.35)	**0.55 (0.42-0.72)**	<0.0001
1-3	66 (55.93)	177 (62.99)	0.74 (0.48-1.15)	0.177	120 (47.06)	313 (58.95)	**0.62 (0.46-0.84)**	0.002	186 (49.87)	490 (60.34)	**0.65 (0.51-0.84)**	0.0007

The results were in bold if the 95% CI excluded 1 or *P*<0.05.

^a^ Adjusted for age and gender.

^b^ Hardy-Weinberg equilibrium (HWE) for combined subjects.

For the case subjects from Guangdong, all three polymorphisms, namely, rs6939340 A allele [AA vs. GG: adjusted OR = 0.55, 95% CI = 0.31-0.96, *P*=0.036; AG/AA vs. GG: adjusted OR = 0.50, 95% CI = 0.37-0.68, *P*<0.0001], rs4712653 T (CT/TT vs. CC: adjusted OR = 0.57, 95% CI = 0.42-0.78, *P*=0.0004) and rs9295536 C allele (AC/CC vs. AA: adjusted OR=0.59, 95% CI=0.43-0.80, *P*=0.0008) were all associated with reduced neuroblastoma risk. When the protective genotypes were combined, the subjects with 1-3 protective genotypes were less likely to develop neuroblastoma than those not carrying the protective genotypes (adjusted OR=0.62, 95% CI=0.46-0.84, *P*=0.002).

To strengthen the conclusion, we further analyzed the association between *CASC15* gene polymorphisms and neuroblastoma risk by combining the study populations from Henan province and Guangdong province. We found that carriers of rs6939340 A allele were associated with decreased neuroblastoma risk (AA vs. GG: adjusted OR=0.50, 95% CI=0.32-0.78, *P*=0.0024; AG/AA vs. GG: adjusted OR=0.56, 95% CI=0.44-0.72, *P*<0.0001; AA vs. GG/AG: adjusted OR=0.64, 95% CI=0.42-0.99, *P*=0.044). Reduced risk was also associated with the rs4712653 T allele (CT/TT vs. CC: adjusted OR=0.65, 95% CI=0.51-0.89, *P*=0.0009) and rs9295536 C allele (AC/CC vs. AA: adjusted OR=0.70, 95% CI=0.54-0.90, *P*=0.0050). Moreover, subjects with 1-3 protective genotypes showed decreased neuroblastoma risk than those without the protective alleles (adjusted OR=0.65, 95% CI=0.51-0.84, *P*=0.0007).

### False positive report probability (FPRP) analysis

The prior probabilities ranging from 0.25 to 0.0001 were used to weigh the significant findings (Table 3). Except for rs6939340 AA vs. GG/AG and 2 protective carriers, all the significant findings remained true when we used a FPRP threshold of 0.2 as suggested by Wacholder *et al.* [29].

**Table 3:** False-positive report probability results for significant findings in combined subjects

Genotype	Crude OR (95% CI)	*P* ^a^	Statistical Power ^b^	Prior probability				
				0.25	0.1	0.01	0.001	0.0001
rs6939340 G>A								
AG vs. GG	0.58 (0.44-0.75)	<0.0001	0.152	**0.001**	**0.002**	**0.024**	**0.199**	0.714
AA vs. GG	0.50 (0.32-0.79)	0.0025	0.108	**0.065**	**0.172**	0.695	0.958	0.996
AG/AA vs. GG	0.56 (0.44-0.72)	<0.0001	0.078	**0.000**	**0.000**	**0.005**	**0.049**	0.339
AA vs. GG/AG	0.64 (0.42-0.99)	0.0451	0.440	0.235	0.480	0.910	0.990	0.999
rs4712653 C>T								
TC vs. CC	0.65 (0.50-0.86)	0.0019	0.446	**0.013**	**0.037**	0.297	0.810	0.977
TC/TT vs. CC	0.65 (0.51-0.84)	0.0009	0.419	**0.006**	**0.019**	**0.175**	0.682	0.955
rs9295536 A>C								
CA vs. AA	0.71 (0.54-0.93)	0.0113	0.678	**0.048**	**0.130**	0.623	0.943	0.994
CA/CC vs. AA	0.70 (0.54-0.90)	0.0049	0.626	**0.023**	**0.066**	0.437	0.887	0.987
2 vs. 0	1.88 (1.02-3.44)	0.0415	0.216	0.366	0.634	0.950	0.995	0.999
3 vs. 0	0.55 (0.42-0.72)	<0.0001	0.120	**0.001**	**0.002**	**0.019**	**0.161**	0.658
1-3 vs. 0	0.65 (0.51-0.84)	0.0007	0.420	**0.005**	**0.015**	**0.142**	0.625	0.943

^a^Chi-square test was used to calculate the genotype frequency distributions.

^b^ Statistical power was calculated using the number of observations in the subgroup and the OR and *P* values in this table.

## DISCUSSION

In the current case-control study, we investigated the potential association between *CASC15* gene polymorphisms and neuroblastoma risk in two distinct populations from China. Given the relative paucity of somatic mutations in high-risk neuroblastoma, the genetic basis of sporadic neuroblastoma still needs to be elucidated. The *CASC15* gene, also referred as *FLJ22536* or *LINC00340* gene spans ∼530 kilobases on chromosome 6p22. It was originally identified *in silico* as a highly active long non-coding RNA (lncRNA) [30]. In 2008, the first GWAS was conducted in the European population to identify common DNA variations that were predisposed to sporadic neuroblastoma [17]. This GWAS consisted of the discovery stage that included 1032 cases and 2043 controls from Europe and the replication stage that included 720 cases and 2128 controls from Northern Europe. Three SNPs were identified in the *CASC15* gene that associated with clinically aggressive neuroblastoma. Since then, these SNPs have been investigated in African-American and Italian populations. Latorre *et al.* studied 390 cases and 2500 controls in an African-American cohort and did not find any association between these 3 *CASC15* gene SNPs with neuroblastoma susceptibility [22]. Capasso *et al.* showed that two *CASC15* gene SNPs (rs6939340 A> G and rs4712653 T>C) were risk factors for neuroblastoma by evaluating 370 cases and 809 controls in an Italian cohort [23]. Mike *et al.* showed that decreased expression of the truncated CASC15-S isoform was associated with more advanced neuroblastoma [31]. We previously conducted a case-control study with 201 cases and 531 controls in southern China and showed that all three SNPs were protective [25]. Moreover, subjects carrying one or more protective genotypes showed lower neuroblastoma susceptibility than non-carriers. However, the findings of our previous study were limited by selection bias and small sample size. Therefore, in this study we investigated the role of the three GWAS-identified SNPs in neuroblastoma risk in the two distinct populations.

The study subjects from Henan showed significant association with neuroblastoma risk only in the rs6939340 G>A variant and not in the rs4712653 C>T and rs9295536 A>C. On the other hand, all three SNPs were associated with decreased neuroblastoma risk in the Guangdong population. Combining the two populations also resulted in similar results. The differences in the relationship between *CASC15* gene polymorphisms and neuroblastoma risk among the two different populations might be ascribed to different environmental exposures as well as different genetic backgrounds. We also found that subjects with 1-3 protective genotypes showed decreased neuroblastoma risk. Further, FPRP analysis excluded the possibility of false positive results in our study, thereby confirming our data. The discrepancy of the role of *CASC15* gene polymorphisms in neuroblastoma risk might be attributed to (1) relative small sample sizes in the validation studies and (2) the variation in allele frequencies, linkage disequilibrium patterns, and environmental exposures among different ethnic populations that influence cancer risk [32].

Although this study is the first to verify the genetic role of *CASC15* gene polymorphisms in two independent populations in China, it has several limitations. First, although this is the largest case-control study conducted to-date, the sample size was still not large enough. The low incidence rate of neuroblastoma in China that makes it challenging to recruit eligible subjects. Second, only three GWAS-identified SNPs in the *CASC15* gene were analyzed and other potential functional SNPs were not tested. Third, the present study focused on the subjects recruited from single hospital rather than from the entire community, which may result in selection bias. Fourth, we only analyzed the genetic factors in neuroblastoma risk, whereas other environmental factors (living environment, dietary intake, and paternal exposures) were not available to conduct gene-environmental interaction analysis.

In summary, our results showed the protective role of three *CASC15* gene polymorphisms in neuroblastoma susceptibility in two independent populations in China. Larger and multicenter-based case-control studies are warranted to further confirm the role of *CASC15* gene polymorphisms in decreasing neuroblastoma risk and determine the molecular mechanism of action.

## MATERIALS AND METHODS

### Study subjects

This study involved two independent case-control populations. The first was derived from our previous study, which was conducted in Guangdong province [25, 26]. The second was conducted in Henan province, which included 118 cases and 281 controls. The recruitment procedure and criteria were as described previously [27, 33]. In brief, we identified 118 histologically confirmed neuroblastoma cases among Henan residents from The First Affiliated Hospital of Zhengzhou University between August 2011 and April 2017. We also recruited 281 control subjects from the same hospital during the same period. The case and control subjects were age- and gender matched. The response rate was approximately 80% for cases and 90% for controls enrolled from Henan province. All subjects or their guardians were informed about the project and gave written consent. This study was approved by the Institutional Review Board of The First Affiliated Hospital of Zhengzhou University.

### SNP selection and genotyping

Three SNPs, rs6939340 G > A, rs4712653 C > T, rs9295536 A > C in the *CASC15* gene were chosen from the previous GWAS study [17]. The genomic DNA was isolated from venous blood samples using TIANamp Blood DNA Kit (TianGen Biotech Co. Ltd., Beijing, China) according to manufacturer’s instructions. Genotyping was performed by Taqman real-time PCR as published previously [26, 33]. To ensure the accuracy of genotyping results, 10% of randomly selected samples were genotyped by DNA sequencing method. The concordance rate for the quality control samples was 100%.

### Statistical analysis

The goodness-of-fit χ^2^ test was used to assess if the selected SNPs deviated from Hardy-Weinberg equilibrium among controls. The two-sided chi-squared test was used to compare demographic variables and genotype frequencies of the cases and controls. ORs and their corresponding 95% CIs were computed by unconditional logistic regression analyses with or without adjusting for age and gender. The FPRP analysis was performed as described previously [29]. All reported *P* values were two sided and a *P* value < 0.05 was considered statistically significant. The SAS statistical package (version 9.1; SAS Institute, Cary, NC) was used to perform all statistical analysis.
